# Locus-Specific Ribosomal RNA Gene Silencing in Nucleolar Dominance

**DOI:** 10.1371/journal.pone.0000815

**Published:** 2007-08-29

**Authors:** Michelle S. Lewis, Diane J. Pikaard, Mikhail Nasrallah, Jed H. Doelling, Craig S. Pikaard

**Affiliations:** 1 Biology Department, Washington University, Saint Louis, Missouri, United States of America; 2 Department of Plant Biology, Cornell University, Ithaca, New York, United States of America; 3 Division of Plant and Soil Sciences, West Virginia University, Morgantown, West Virginia, United States of America; University of Munich, Germany

## Abstract

The silencing of one parental set of rRNA genes in a genetic hybrid is an epigenetic phenomenon known as nucleolar dominance. We showed previously that silencing is restricted to the nucleolus organizer regions (NORs), the loci where rRNA genes are tandemly arrayed, and does not spread to or from neighboring protein-coding genes. One hypothesis is that nucleolar dominance is the net result of hundreds of silencing events acting one rRNA gene at a time. A prediction of this hypothesis is that rRNA gene silencing should occur independent of chromosomal location. An alternative hypothesis is that the regulatory unit in nucleolar dominance is the NOR, rather than each individual rRNA gene, in which case NOR localization may be essential for rRNA gene silencing. To test these alternative hypotheses, we examined the fates of rRNA transgenes integrated at ectopic locations. The transgenes were accurately transcribed in all independent transgenic *Arabidopsis thaliana* lines tested, indicating that NOR localization is not required for rRNA gene expression. Upon crossing the transgenic *A. thaliana* lines as ovule parents with *A. lyrata* to form F1 hybrids, a new system for the study of nucleolar dominance, the endogenous rRNA genes located within the *A. thaliana* NORs are silenced. However, rRNA transgenes escaped silencing in multiple independent hybrids. Collectively, our data suggest that rRNA gene activation can occur in a gene-autonomous fashion, independent of chromosomal location, whereas rRNA gene silencing in nucleolar dominance is locus-dependent.

## Introduction

Genes that encode the precursor transcript for the three largest ribosomal RNAs (18S, 5.8S and 25S rRNAs) are present in hundreds to thousands of copies in eukaryotic genomes [1], [2], [3]. Clustered in loci that span millions of base pairs, the tandem arrays of rRNA genes are known as nucleolus organizer regions (NORs) because their expression brings about the formation of the nucleolus where ribosome assembly takes place [4], [5], [6], [7]. For this reason, the term “nucleolar dominance” [8] has been used to describe the expression of NORs inherited from only one progenitor in a plant or animal hybrid [9], [10], [11], [12], [13], [14]. Nucleolar dominance is among the most extensive gene silencing phenomena known, second in scope only to X-chromosome inactivation in female mammals [15], [16]. Unlike X-inactivation in somatic cells, the choice of which set of rRNA genes to silence is not random but instead involves silencing of rRNA genes inherited from the same progenitor, independent of maternal or paternal effects.

Several lines of evidence implicate the chromatin environment of rRNA genes in the control of nucleolar dominance. In Brassica or Arabidopsis allopolyploid hybrids, underdominant rRNA genes (the set of rRNA genes that is subject to silencing) transfected into protoplasts are transcribed even though their endogenous, chromosomal counterparts are silenced [17], [18]. These experiments indicate that the necessary transcription factors are available in the cell but that chromosomal genes are denied access to them. The repression of the silenced chromosomal rRNA genes is mediated by chromatin modifications that include cytosine methylation, histone deacetylation and histone H3 lysine 9 dimethylation and can be reversed by chemical inhibitors of DNA methylation or histone deacetylation [18], [19], [20] or by genetic knockdown of specific chromatin modifying activities, such as histone deacetylases *HDT1* and *HDA6* [21], [22].

The genic or chromosomal signals that are required for ribosomal gene silencing in nucleolar dominance are unknown. One clue is that in *Arabidopsis suecica*, the allotetraploid hybrid of *A. thaliana* and *A. arenosa*, silencing of the *A. thaliana*-derived rRNA genes is restricted to the NORs and does not spread to or from adjacent protein coding genes [23]. One hypothesis is that NOR inactivation is simply the summation of hundreds of silencing events, with each individual rRNA gene being an independent unit of regulation. A prediction of this hypothesis is that rRNA gene silencing should occur regardless of an rRNA gene's chromosomal location or copy number. An alternative hypothesis is that the NOR is the unit of regulation [24] in which case rRNA genes may need to be localized within an NOR in order to be silenced. In *Arabidopsis thaliana* there are two NORs, *NOR2* and *NOR4*, each of which is composed of ∼375–400 rRNA genes arranged head-to-tail in an uninterrupted array spanning ∼4 Mbp [25]. NOR2 and NOR4 are located at the northern tips of chromosomes 2 and 4, respectively, where they are capped by ∼2.5–3 kb of consensus telomere repeats [26], [27]. The large sizes and distal positions of the NORs could contribute to a unique chromatin environment at these loci.

We examined the chromosomal parameters affecting nucleolar dominance in F1 hybrids of *Arabidopsis thaliana*×*Arabidopsis lyrata* [28], a new hybrid system we have developed for the study of nucleolar dominance and report here for the first time. In *A. thaliana*×*A. lyrata* hybrids, the *A. thaliana* rRNA genes are silenced and the *A. lyrata* rRNA genes are expressed. We show that full-length *A. thaliana* rRNA transgenes integrated into the *A. thaliana* genome can escape silencing in hybrids in which the endogenous *A. thaliana* rRNA genes located within the NORs are subjected to nucleolar dominance and are repressed. These results argue against the hypothesis that individual rRNA genes are the units of regulation in nucleolar dominance. Instead, the data support the hypothesis that chromosomal position, extensive rRNA gene redundancy, or a local chromatin environment typified by NORs is important for uniparental rRNA gene silencing.

## Results and Discussion

### Generation of transgenic *Arabidopsis thaliana* bearing ectopic rRNA genes

**Figure 1:**
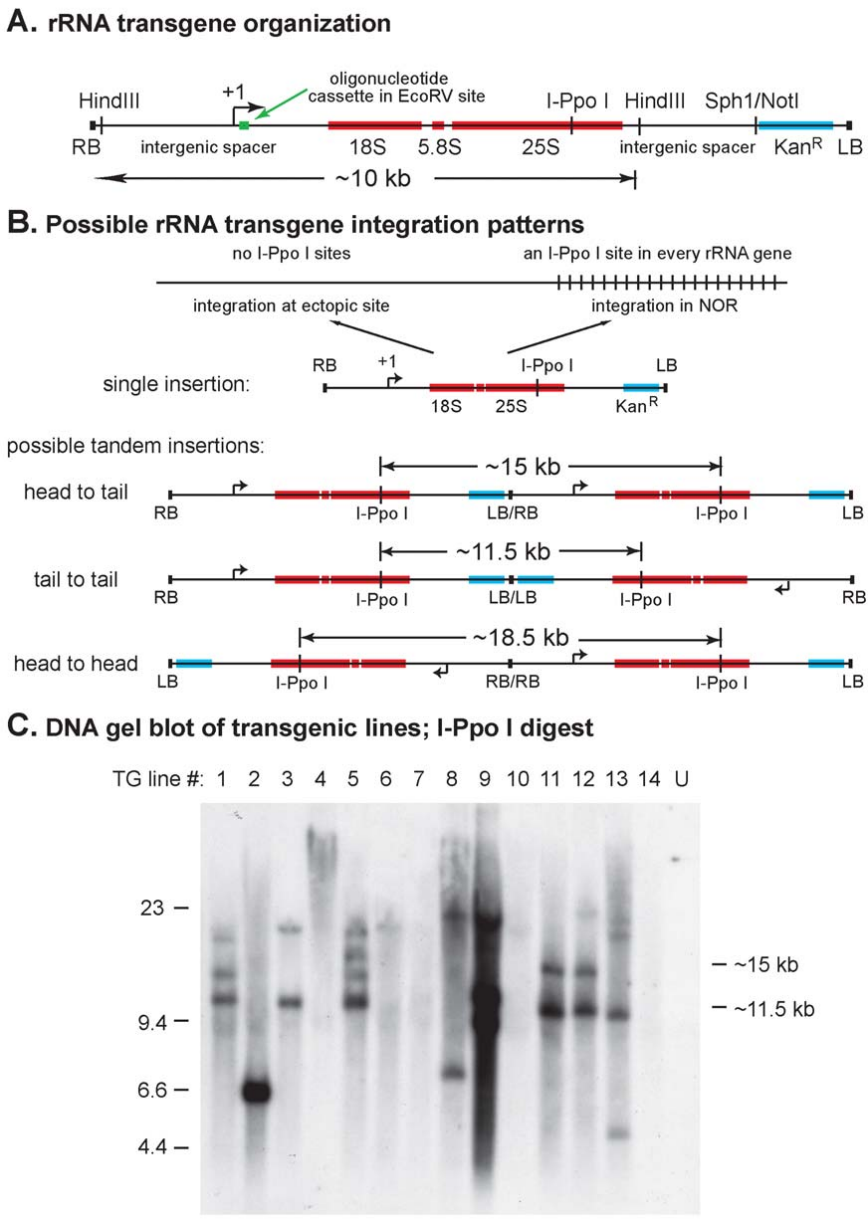
Arabidopsis rRNA transgene construct and DNA blot of transgenic lines. A. Map of the T-DNA construct used to generate transgenic plants. RB and LB indicate right and left borders of the transferred DNA (T-DNA), respectively. The arrow denoted by +1 indicates the transcription initiation site at the gene promoter; this site is 92 bp upstream of a 19 bp oligonucleotide cassette inserted into the rRNA gene sequences. The coding regions of the 18S, 5.8S and 25S rRNAs are depicted as red rectangles, and the kanamycin resistance gene as a light blue rectangle. A complete, unit-length rRNA gene is ∼10.5 kb in size. The transgene is larger than unit length due to the presence of intergenic spacer sequences downstream, as well as upstream, of the coding sequences. B. Possible single and tandem transgene integration patterns expected for DNA blots following digestion of genomic DNA with I-Ppo I and probing with the kanamycin resistance gene. Note that only I-Ppo I fragments containing the kanamycin resistance gene will be detected on the Southern blot, thus the 18.5 kb fragment of a head-to-head tandem insertion would not be detected. C. DNA blot of genomic DNA from transgenic lines following digestion with I-Ppo I and detection with a ^32^P-labeled, kanamycin resistance gene probe. The digested fragments were size-separated on a 0.3% agarose gel. Bacteriophage Lambda DNA digested with HindIII served as a size ladder for the estimation of fragment lengths.

To determine if rRNA genes must be localized within an NOR to be properly expressed or subjected to nucleolar dominance, *A. thaliana* rRNA transgenes were stably integrated into the *A. thaliana* genome by Agrobacterium-mediated transformation [29]. The T-DNA (transferred DNA) construct consisted of complete rRNA coding sequences flanked on both sides by intergenic spacer sequences so as to include any potential elements required for transcriptional activation or termination (Fig. 1A). To probe the nature of the insertions, Southern blot analysis with a T-DNA-specific probe (kanamycin resistance gene) was performed following digestion of genomic DNA with the intron-encoded homing endonuclease I-PpoI. This enzyme is encoded within a mobile group I intron, located within the 25S coding sequences of *Physarum polycephalum*, that can spread to rRNA genes lacking the intron as a result of a gene conversion mechanism initiated by I-PpoI cleavage [30]. The I-PpoI cleavage site is highly conserved in evolution, thus I-PpoI will cut each Arabidopsis rRNA gene within the 25S coding sequence but does not appear to cut elsewhere in the genome; notably its 15 bp recognition sequence would occur, by chance, only once every billion basepairs (equivalent to seven *A. thaliana* genomes) [27].

An I-Ppo I recognition site is present in the rRNA transgene construct (see Fig. 1A), therefore a single copy of the transgene integrated at an ectopic site would be cut by I-PpoI to yield flanking fragments too large to resolve on a 0.3% agarose gel (Fig. 1B). By contrast, a T-DNA integrated within the NORs on chromosomes 2 or 4 would yield I-PpoI fragments of 6–16 kb, depending on the distance from the site of integration to the I-PpoI sites of the flanking endogenous rRNA genes. Head-to-tail (right border/left border junction; RB/LB) or tail-to-tail (LB/LB) tandem insertions at ectopic locations would yield I-PpoI fragments of ∼15 kb or ∼11.5 kb, respectively using the kanamycin resistance (Kan^r^) gene as the probe for DNA blot analysis. A head-to-tail insertion would also yield an additional unresolved large DNA fragment including the Kan^r^ gene. Tandem head to head copies are expected to yield a ∼18 kb I-PpoI fragment lacking the Kan^r^ gene as well as two large Kan^r^ -containing DNA fragments.

DNA fragments consistent with the size predictions above are apparent in the DNA blot shown in Fig. 1C. In this experiment, genomic DNA of 14 independent transgenic lines was digested with I-PpoI and probed with the Kan^r^ gene. During the process of T-DNA transfer from *Agrobacterium tumefaciens* to plants, T-strand synthesis typically initiates at the right border sequence but can terminate short of, at or beyond the left border element [31]. As a result, some variation in restriction fragment size can be expected when comparing independent transgenic lines on Southern blots. Bands of approximately 15 kb and/or 11.5 kb are present in many of the lines (Lines 1, 3, 5, 9,11, 12, 13), indicative of two or more transgenes that integrated head-to-tail or tail-to-tail, respectively. In some lines (e.g. lines 2, 8, 9 and 13), additional bands were detected that were consistent with possible T-DNA integration into one of the NORs but subsequent genetic mapping showed that this was not the case (discussed further in a later section and in the supplemental data). Collectively, our analyses of the transgenic lines indicate that they typically contained multiple tandem copies of the transgene, integrated at ectopic locations in a variety of orientations.

### NOR-independent rRNA transgene expression

**Figure 2:**
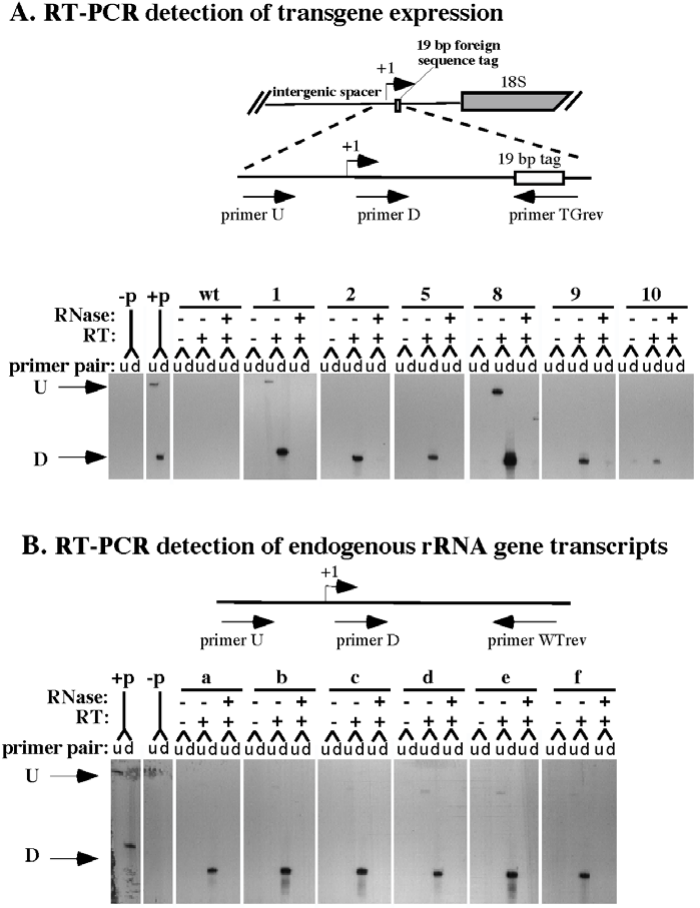
rRNA transgenes are expressed in *A. thaliana.* A. RT-PCR analysis of rRNA transgene activity in six independent transgenic lines. The diagram shows the location of primers used to detect transgene expression. A unique 19 bp tag located 92 bp downstream of the transcription start site distinguishes transgene transcripts from endogenous rRNA transcripts. RNA from wild-type (wt) or transgenic plants was treated with reverse transcriptase (RT), or was treated with RNase prior to reverse transcriptase as a control. Two equal aliquots of from each treatment were then subjected to PCR using primer pairs to detect transcripts downstream (D) or upstream (U) of the expected transcription start site, with the TGrev primer being end-labeled with ^32^P. Reaction products were run in adjacent lanes of the gel and visualized by autoradiography. PCR using rRNA gene plasmid DNA (p) served as controls for the D and U PCR products. Stronger D than U signals suggest that transcripts are initiated properly from the rRNA gene promoter. U signals result from transcripts that read-through the promoter region from upstream. B. RT-PCR analysis of endogenous rRNA gene transcripts in six independent transgenic plants.

A 19 bp foreign sequence tag inserted into the rRNA gene sequence at +92 (Fig. 2A) allowed us to discriminate transgene transcripts from endogenous rRNA transcripts (Fig. 2B). Using RT-PCR with an end-labeled transgene-specific reverse primer (TGrev) and a forward primer corresponding to sequences immediately downstream of the pol I transcription start site (primer D in Fig. 2A), transgene transcripts were detected in all transgenic plants tested (PCR product D in Fig. 2A), but not in wild-type (wt) control (non-transgenic) plants. A second forward primer which anneals 90 bp upstream of the transcription start site (primer U) was used as a control to test for transcripts that might originate upstream of the gene promoter and read-through the promoter region [32]. Using plasmid DNA containing a cloned rRNA gene as template, the upstream (U) and downstream (D) PCR products were generated with similar efficiency (Fig 2A, +p control). However, only trace amounts of upstream RT-PCR product were detected in all transgenic lines except for Line 8 in which a strong U (upstream) signal was obtained, paired with an extremely strong D (downstream) signal (Fig. 2A). A similar RT-PCR strategy to detect transcripts upstream and downstream of the promoters of endogenous rRNA genes revealed strong transcription signals downstream of the promoter and trace amounts of transcripts reading through the promoter from upstream (Fig. 2B), similar to the transcription signals detected for the transgenes. Collectively, our data indicate that the rRNA transgenes are expressed in a manner indistinguishable from the endogenous rRNA genes and that the different transgene integration patterns detected by Southern blotting have no discernible influence on transgene expression.

The detection of strong downstream signals, coupled with weak upstream signals, in Figure 2 indicates that transcripts are initiated within the ∼100 bp interval between the upstream and downstream forward primers, which includes the transcription initiation site, defined as +1 [32]. Because no transcription initiation sites other than the +1 site have been identified within this 100 bp window by transient expression studies or analyses of endogenous rRNA genes [32], [33], we conclude that the transgene promoters are accurately recognized by RNA polymerase I. An earlier study by Wanzenbock et al. also came to the conclusion that ectopic rRNA transgenes in Arabidopsis are likely transcribed by RNA polymerase I [34]. Therefore, although the ∼750–800 endogenous rRNA genes are arrayed head-to-tail at NOR2 and NOR4 [25], [26], [27], the transcription analyses, coupled with the mapping of transgene integration sites, show that rRNA genes do not have to be localized within an NOR in order to be transcribed, in agreement with initial studies in Drosophila [35]. The trace amounts of transcript that read through the promoter may be generated from the spacer promoters that are located at ∼1 kb intervals upstream of the gene promoter [32], from adjacent genes, or from cryptic promoters.

### Nucleolar dominance in *A. lyrata*×*A. thaliana* hybrids

**Figure 3:**
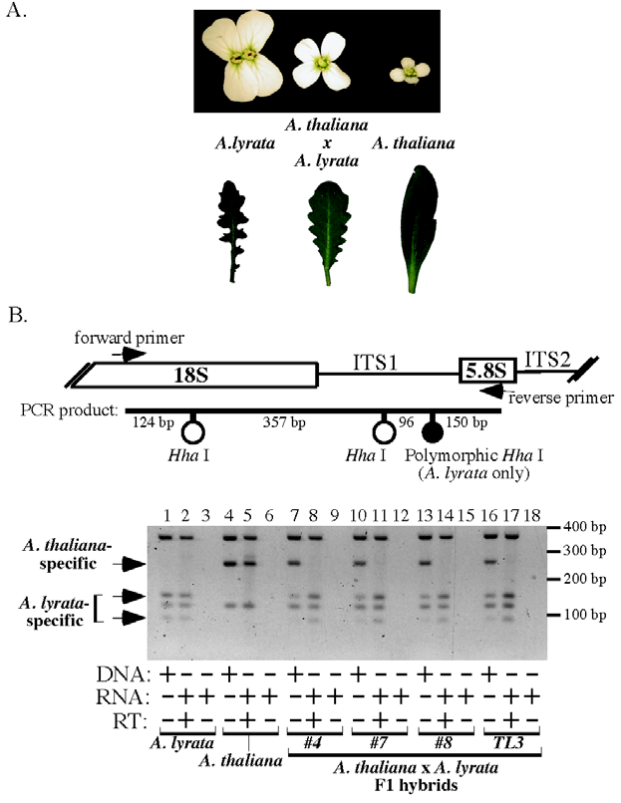
Nucleolar dominance in *A. thaliana×A. lyrata* F1 hybrids. A. *A. thaliana×A. lyrata* hybrids display morphological features intermediate between those of the parents, including flower size and leaf shape. Leaf photos are not to the same scale. B. Detection of rRNA genes and transcripts in *A. thaliana×A. lyrata* hybrids. The diagram shows the intervening sequence 1 (ITS1) region, the location of PCR primer sites within 18S and 5.8S rRNA coding sequences and the *Hha* I sites used to discriminate genes and transcripts of *A. thaliana* from those of *A. lyrata*. PCR using genomic DNA (lanes 1, 4, 7, 10, 13, 16) verifies that rRNA genes of the parental species are present in the hybrids (lanes 7, 10, 13, 16). Products resulting from reverse transcription (RT) of purified RNA followed by PCR and *Hha* I digestion were subjected to electrophoresis in lanes 2, 5, 8, 11, 14, 17. Controls in which RT was omitted prior to PCR are represented by lanes 3, 6, 9, 12, 15, 18. The lack of products in the latter controls indicates that RNA samples are free of contaminating genomic DNA. Note that the *A. lyrata×A. thaliana* F1 hybrids express *A. lyrata* but not *A. thaliana* rRNA genes (lanes 8, 11, 14, 17) in all independent F1 hybrids (four are shown).

*A. lyrata* can be crossed with *A. thaliana* [28], resulting in F1 hybrids whose morphological characters are typically intermediate between those of the parents (Fig. 3A). However, analysis of rRNA gene expression in these hybrids revealed that the rRNA genes of the two progenitors are not co-dominant. As shown for four independent hybrids in Figure 3B, the hybrids inherit rRNA genes of both *A. thaliana* and *A. lyrata* (lanes 7, 10, 13, 16), but only the *A. lyrata* rRNA gene transcripts are readily detected (lanes 8, 11, 14, 17) using an RT-PCR assay that exploits a polymorphism for the restriction endonuclease *Hha*I (compare hybrids to the *A. thaliana* and *A. lyrata* controls in lanes 1–6). By contrast, *A. thaliana* rRNA transcripts are essentially undetectable, showing that nucleolar dominance occurs and that the *A. thaliana* rRNA genes are the underdominant set of genes in the hybrids.

### Locus-dependent rRNA transgene silencing in hybrids

To test the sensitivity of rRNA transgenes to silencing in hybrids, we chose three *A. thaliana* lines displaying distinct patterns of transgene organization (refer to Fig. 1) but similar levels of transgene expression, namely Lines 1, 2, and 9. These lines were chosen for three reasons. First, all three of these lines displayed strong transgene expression initiating from the promoter region and little readthrough transcription coming from upstream (Figure 3A). Second, Southern blot analysis of Lines 1 and 9 indicated the presence of rRNA transgene clusters that likely included transgenes organized tail-to-tail as well as head-to-tail; the two lines nonetheless had unique fragment patterns. Third, the I-PpoI endonuclease fragments smaller than ∼11 kb in Lines 2 and 9 suggested that the transgenes in these lines might have integrated into one of the two NORs, which made them of special interest early in the study. However, late in the study this latter possibility was ultimately ruled out by cloning and sequencing the junctions between the T-DNA and flanking genomic DNA and by showing that the junctions co-segregated with the Kan^r^ resistance marker but did not map to either NOR (see Supporting Information Text S1 and Figure S1).

**Figure 4:**
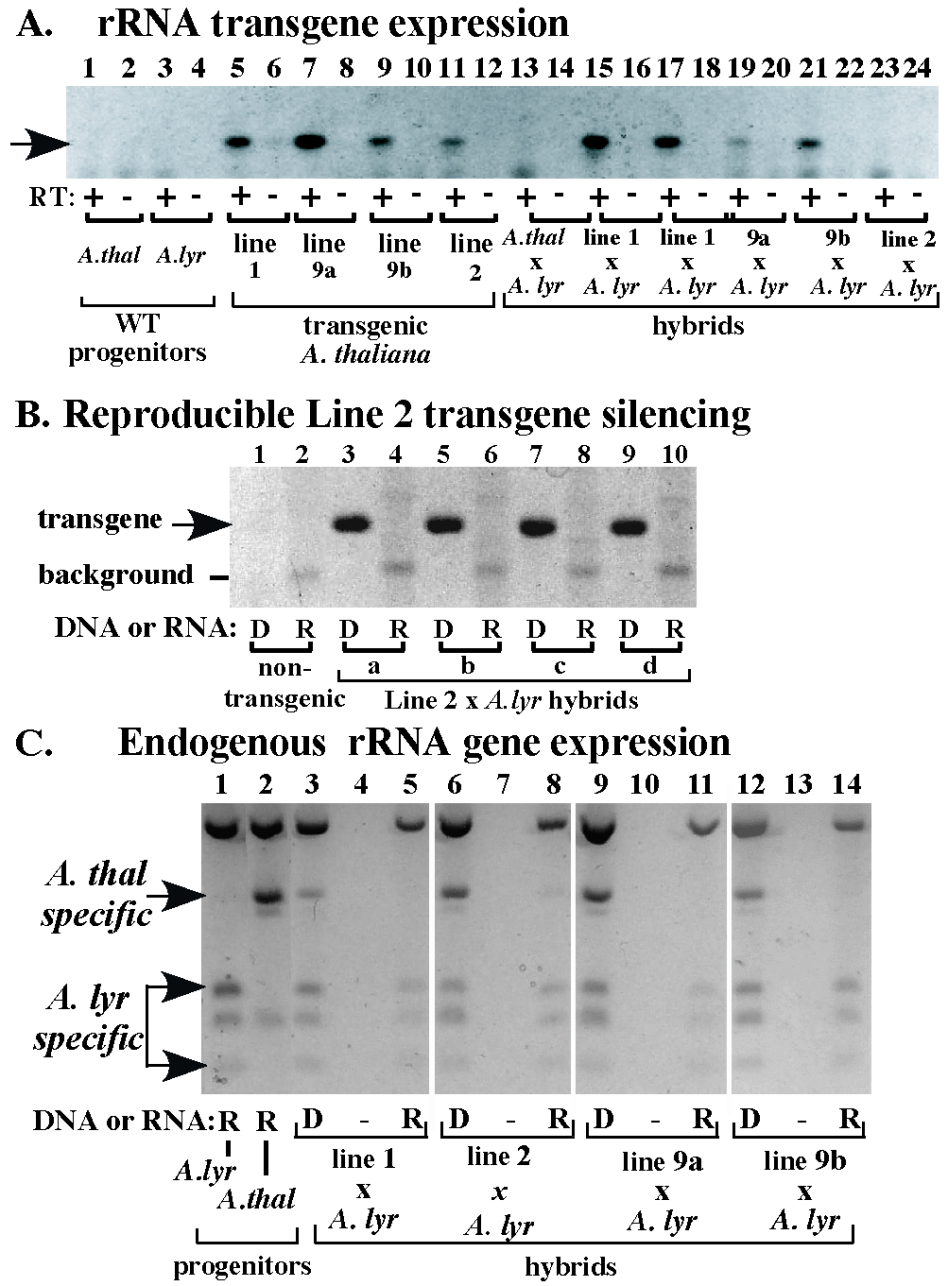
Locus-dependent rRNA transgene expression in *A. thaliana×A. lyrata* hybrids. A. Detection of transgene transcripts using RT-PCR and a transgene-specific primer pair. Transgene transcripts were detected in T3 plants of *A. thaliana* transgenic Line 1, Line 9 (two lines descended from different T2 sibs; 9a and 9b), and Line 2 (lanes 5–12). No RT-PCR products were detected using RNA of wild-type *A. thaliana* or *A. lyrata* (lanes 1 and 3) or wild-type hybrids (lane 13), confirming the transgene specificity of the PCR assay. Hybrids formed using transgenic *A. thaliana* lines were analyzed in lanes 15–24. Two sibling Line 1×*A. lyrata* hybrids were analyzed in lanes 15–18. Note that transgene transcripts were detected in all hybrids except the hybrid derived from Line 2. B. Line 2 transgenes are present in hybrids, but are consistently silenced. DNA or RNA of non-transgenic *A. thaliana* (ecotype Landsberg) and four independent Line 2×*A. lyrata* hybrids was tested by PCR or RT-PCR, respectively. Note that the transgenes are present in the hybrids (see DNA control lanes, labeled D) but are not expressed into RNA (R lanes). C. Endogenous *A. thaliana* rRNA genes are silenced in transgenic hybrids. RT-PCR was conducted as in Figure 3B using RNA of the progenitor species (lanes 1 and 2), and of hybrids resulting from crossing *A. lyrata* with transgenic Lines 1, 4, and 9 (lanes 5, 8, 11, 14). PCR controls using genomic DNA as template shows that all hybrids inherited rRNA genes from both parents (lanes 3, 6, 9, 12), although only *A. lyrata* rRNA genes are expressed.

Transgenic plants for lines 1, 2 and 9 (T1 generation) were selfed for two generations and homozygous T3 lines were identified based on the segregation of the kanamycin resistance marker. RT-PCR analysis confirmed that in each line rRNA transgenes were still expressed in the T3 generation (Fig. 4A, lanes 5, 7, 9, 11) and thus showed no evidence of silencing due to RNA interference (RNAi) or cosuppression-like phenomena. No transcripts were detected using transgene-specific primers to amplify non-transgenic *A. thaliana* or *A. lyrata* controls, as expected (Fig. 4A, lanes 1 and 3). Next, T3 individuals of Lines 1, 2, and 9 were crossed with *A. lyrata* to form F1 hybrids. Importantly, the *A. thaliana* transgenes in Lines 1 and 9 were not silenced in the hybrids (Fig. 4A, lanes 15, 17, 19, 21), despite the silencing of the ∼750–800 endogenous, NOR-localized *A. thaliana* rRNA genes in these lines (Fig. 4C, lanes 5, 8, 11, 14). However, the rRNA transgenes of Line 2 were silenced in the hybrid (Fig. 4A, lane 23) and this was true in four independent Line 2×*A. lyrata* hybrids (Fig. 4B).

Collectively, our data show that rRNA genes stably integrated at ectopic locations can escape nucleolar dominance (Lines 1 and 9), indicating that the mechanisms of nucleolar dominance do not silence individual rRNA genes, or small clusters of rRNA genes, independent of their genomic location. Instead, we deduce that rRNA gene silencing only occurs at selected loci, such as the NORs. The data also indicate that chromosomal loci in addition to NORs might be compatible with the silencing of ectopic rRNA genes, as suggested by the highly reproducible silencing of the rRNA transgenes carried by Line 2 upon hybrid formation. We are cautious about the latter interpretation, however, because it is based on a negative result - the lack of rRNA gene expression. Although it is possible that the observed Line 2 rRNA transgene silencing is due to nucleolar dominance-related mechanisms, potential transgene silencing mechanisms unrelated to nucleolar dominance cannot be ruled out. At the present time, we are unable to distinguish between these possibilities in the relatively unexplored *A. thaliana×A. lyrata* hybrid system. Moreover, the transgene(s) in Line 2 appears to have undergone dramatic rearrangement or internal deletion in order to explain the small (∼6.6 kb) I-PpoI fragment detected on the Southern blot in Figure 1 without having integrated into an NOR (Figure S1). Therefore it is possible that the T-DNA of Line 2 might have lost sequences that would have protected or insulated the rRNA gene promoters from the influence of the surrounding chromatin environment. In this regard it may be significant that the rRNA transgene cluster in Line 2 integrated in a transposon-rich region that is expected to be predominantly heterochromatic. Nonetheless, it is intriguing that the silencing of the Line 2 transgenes only occurred in the context of the hybrid and not in three generations of *A. thaliana* inbreeding. If the potential to form heterochromatin in response to hybridization is key to rRNA gene silencing in nucleolar dominance, NORs may share this property with numerous loci distributed throughout the genome.

## Materials and Methods

### Plant growth and interspecies hybridization

*A. lyrata* was grown in the greenhouse and served as the pollen donor for all hybrids [28]. *A. thaliana* (ecotype Landsberg) flower buds were emasculated and hand-pollinated. Hybrids were grown in a growth chamber (8 hr light, 16 hr dark, 21°C).

### rRNA transgene construction and transformation

An *A. thaliana* rRNA gene flanked on each side by intergenic spacers (from the HindIII site at position −2590 upstream of the transcription initiation site in the first intergenic spacer through the entire rRNA coding region to the SphI site at position −110 relative to the transcription initiation site in the next intergenic spacer) was cloned between the HindIII and NotI sites of pMON10098 (http://www.hos.ufl.edu/kleeweb/pmon10098.htm). A double-stranded oligonucleotide cassette of sequence 5′-GTAGGCCAGCTGGGCCATG-3′ was inserted into the *Eco*RV site at +92 of the first intergenic spacer. *A. thaliana* (Landsberg erecta) was transformed using Agrobacterium and the vacuum infiltration technique [29]. Transformants (T1 generation) were selected on sterile germination medium [36] containing 75 mg/L kanamycin. Homozygotes (T3 generation) were crossed with *A. lyrata* to form F1 hybrids.

### Nucleic acid isolation, Southern blot hybridization, and reverse transcription

Plant tissue was frozen in liquid nitrogen, ground to a powder, and mixed vigorously with 3 volumes (w/v) extraction buffer (250 mM Tris-HCl, pH 8.5, 375 mM NaCl, 25 mM EDTA, 1% (w/v) SDS, 1% (v/v) 2-mercaptoethanol). The homogenate was extracted with phenol/chloroform, and total nucleic acids were precipitated from the aqueous phase with isopropanol. After centrifugation, pellets were resuspended in sterile water and total RNA was precipitated with 2 M LiCl. Genomic DNA in the supernatant was recovered by ethanol precipitation.

Genomic DNA digested with the restriction enzyme I-PpoI was size separated on a 0.3% agarose gel prior to blotting onto a nitrocellulose membrane. A 1.3 kb DNA fragment containing the kan^R^ gene, labeled by nick translation using *E. coli* DNA polymerase I and α-^32^P dATP, served as probe for DNA blot hybridization.

Total RNA to be used in reverse transcription (RT) PCR was digested with RQ1 DNase (Promega) to eliminate contaminating genomic DNA. RT reactions contained 1–2.5 ug RNA, 120 ng random hexamers, 0.5 mM dNTPs, and 200 units of reverse transcriptase (Superscript II, Invitrogen) in a 10–20 ul reaction.

### RT-PCR assay of rRNA transgene and endogenous gene transcripts in *A. thaliana*

RT-PCR to detect transgene transcripts was performed using upstream (U) 5′-CGGGGTACCTTCCAAGTATTTCTTTTTTTTTGGC-3′ and downstream (D) 5′-GTGTTGAGGGAGTCTGGGCAGTCCGT-3′ forward primers in combination with the ^32^P-5′ end-labeled (using T_4_ polynucleotide kinase and γ-^32^P-ATP) reverse primer (TGrev) 5′-TCATTCCTCGTGTCGATCATGGCCCAGCT-3′. The underlined nucleotides of the TGrev primer are complementary to the oligonucleotide cassette inserted within the EcoRV site of the transgene. PCR amplification was conducted for 30 cycles of 94°C, 30 sec; 60°C; 30 sec, and 72°C, 60 sec. Products were subjected to electrophoresis on a 6% denaturing polyacrylamide gel and visualized by autoradiography. Plasmid DNA was used as a control to identify amplified fragments of the appropriate size. RT-PCR detection of endogenous rRNA transcripts was conducted in the same way except that only 20 cycles of PCR was performed and a different reverse primer was utilized: (WTrev) 5′-GGACGGTCGGTCATTCCTCGTGTCGAT-3′.

### PCR assays in hybrids

PCR amplification of the ITS1 region of the endogenous rDNA genes (control reactions) used ∼100 ng of genomic DNA in a 50 ul reaction with 20 pmols of each the ITS1 forward primer 5′-GCGCTACACTGATGTATTCAACGAG-3′ and the ITS1 reverse primer 5′-CGCACCTTGCGTTCAAAGACTCGA-3′. RT-PCR amplification of endogenous gene transcripts used DNA generated from 50–200 ng of input RNA rather than genomic DNA. The reactions were allowed to proceed 29 cycles (94°C, 30 sec; 59°C, 30 sec; 72°C, 60 sec). The amplified PCR products were digested with *Hha*I to differentiate between the *A. thaliana* and *A. lyrata* products. For RT-PCR detection of the rRNA transgenes in hybrids, 40 cycles (94°C, 30 sec; 65°C, 30 sec; 72°C, 30 sec) were performed with the TGrev primer and the forward 5′-AGGGGGGTGGGTGTTGAGGGA-3′ primer. Products were resolved on 2% agarose gels in TBE buffer (50 mM Tris-HCl, 50 mM borate, 1mM EDTA, pH 8.3). The gels were stained with ethidium bromide and gel images were obtained by digital photography.

## Supporting Information

Figure S1 Transgene integration sites in *A. thaliana* lines 1 and 2. Stars indicate the location of the transgene clusters. Red arrows represent predicted genes without associated ESTs, green arrows represent active genes for which ESTs exist, and black arrows represent transposon-related repetitive elements. A. The transgene cluster in Line 1 is located on chromosome 1 in a region where genes are predicted once every 4 kb, on average. The majority of the genes in this region are expressed. B. The transgene cluster in Line 2 is located on chromosome 5 in a region of low gene density in which LINE and Ty1/copia-like transposable element sequences are the predominant feature. Although *Arabidopsis thaliana* averages one gene every 5 kb, predicted genes in this region occur, on average, once every 10 kb. Also of note in this region, ∼5 kb to the left of the transgene cluster, is an ∼600 bp sequence composed of a tandemly repeated 27 bp element. The figure was drawn with the aid of the TAIR Mapviewer tool (www. arabidopsis.org) and TIGR (www.tigr.org) annotation data.
(0.41 MB TIF)

Text S1 Identification of transgene integration sites in Lines 1, 2 and 9.
(0.05 MB DOC)
