# Supplementary figures and images for: Locus-Specific Ribosomal RNA Gene Silencing in Nucleolar Dominance
Source: PLoS One. 2007 Aug 29;2(8):e815. doi: 10.1371/journal.pone.0000815 (PMC1950575; doi:10.1371/journal.pone.0000815)

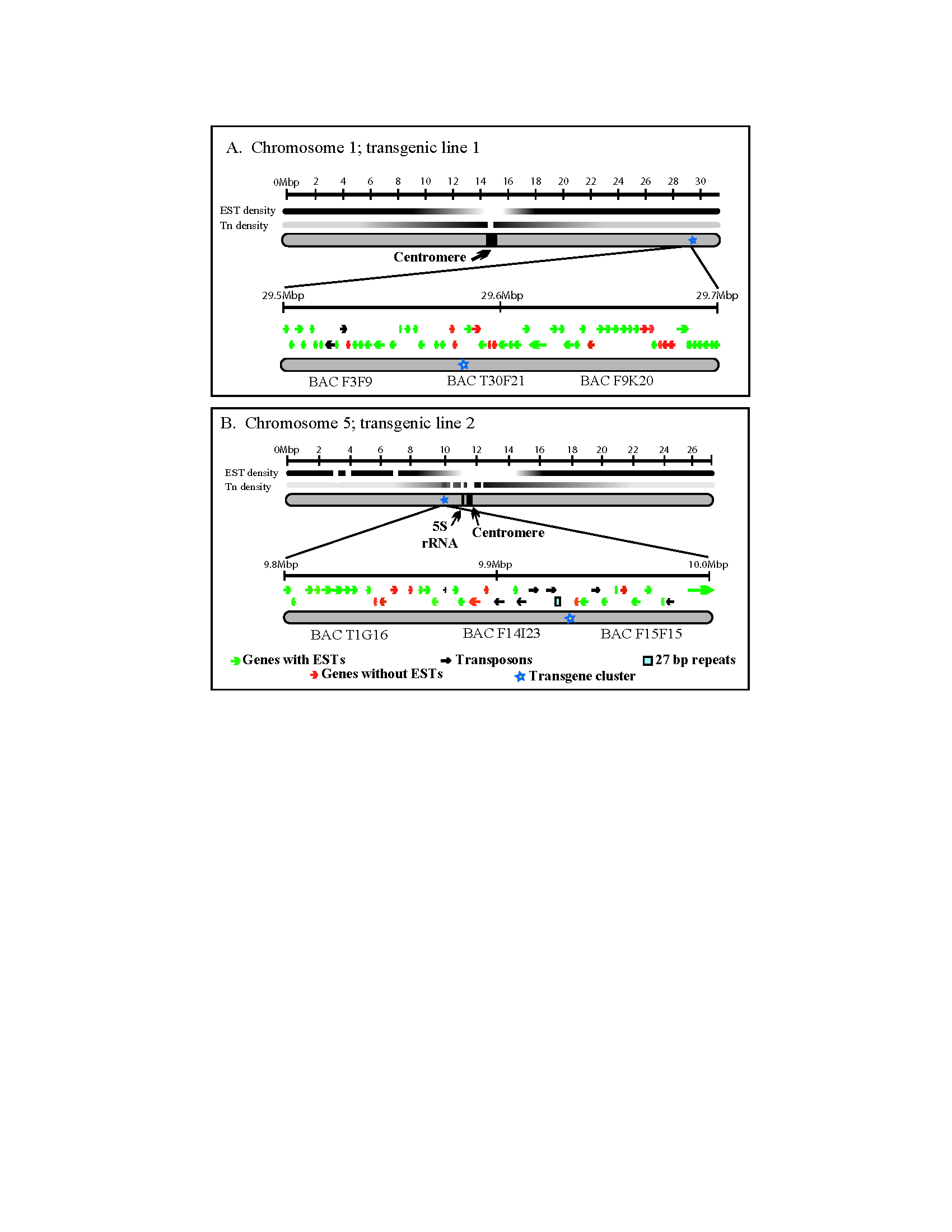

Supplement: Figure S1 — Transgene integration sites in A. thaliana lines 1 and 2. Stars indicate the location of the transgene clusters. Red arrows represent predicted genes without associated ESTs, green arrows represent active genes for which ESTs exist, and black arrows represent transposon-related repetitive elements. A. The transgene cluster in Line 1 is located on chromosome 1 in a region where genes are predicted once every 4 kb, on average. The majority of the genes in this region are expressed. B. The transgene cluster in Line 2 is located on chromosome 5 in a region of low gene density in which LINE and Ty1/copia-like transposable element sequences are the predominant feature. Although Arabidopsis thaliana averages one gene every 5 kb, predicted genes in this region occur, on average, once every 10 kb. Also of note in this region, ∼5 kb to the left of the transgene cluster, is an ∼600 bp sequence composed of a tandemly repeated 27 bp element. The figure was drawn with the aid of the TAIR Mapviewer tool (www. arabidopsis.org) and TIGR (www.tigr.org) annotation data. (0.41 MB TIF) [file pone.0000815.s001.tif]
