# Supplementary material for: Locus-Specific Ribosomal RNA Gene Silencing in Nucleolar Dominance
Source: PLoS One. 2007 Aug 29;2(8):e815. doi: 10.1371/journal.pone.0000815 (PMC1950575; doi:10.1371/journal.pone.0000815)
Supplement: Text S1 — Identification of transgene integration sites in Lines 1, 2 and 9. (0.05 MB DOC) [file pone.0000815.s002.doc]

**Supporting information**

**Identification of transgene integration sites in Lines 1, 2 and 9.**

To determine the genomic locations of rRNA transgene clusters in selected lines showing distinct patterns of integration on Southern blots (Lines 1, 2 and 9; refer to Figure 1 of the paper), junctions between T-DNA and flanking genomic DNA were cloned following inverse PCR. Recovered sequences were then confirmed by using junction PCR products as molecular markers to show that the junctions co-segregated with the Kan^r^ gene in an F2 mapping population.

For inverse PCR, purified genomic DNA of *A. thaliana* transgenic Lines 2 and 9 was digested overnight with *Nsi* I; Line 1 was digested with *Nhe* I. Following dilution and ligation to allow linear fragments to circularize, inverse PCR was performed. All reactions used the inverse PCR-forward primer 5’ GTAGATTTCCCGGACATGAAGC 3’. The reverse primer for Lines 2 and 9 was 5’ GTATAATTGCGGGACTGTAATC 3’. For Line 1, the reverse primer was 5’ TGATGGCATTTGTAGTGCCAC 3’. Resulting PCR products were sequenced using an ABI 3700 automated sequencer and big dye terminator technology. Chromosomal locations of recovered sequences were determined using a BLASTn search of the *A. thaliana* genome.

To verify that the PCR products represented true junctions between genomic DNA and T-DNA, transgenic lines (ecotype Landsberg erecta) were crossed to wild type plants (ecotype Columbia) and an F_2_ mapping population was generated. F2 individuals were scored for the presence of the T-DNA-encoded kanamycin resistance gene and for T-DNA/genomic DNA junctions at the T-DNA left border using PCR. The junction-specific molecular marker was generated using the inverse PCR-forward primer in combination with specific primers for each transgene based on flanking sequences recovered by inverse PCR. For transgenic Line 1, the primer was 5’ CGAAACAACGGCCCATACCTAG 3’, for Line 2: 5’ GTCACAACTTTCACGCGCAAAAC 3’, and for Line 9: 5’ ATGCATCGTCCGAAACCAATTG 3’. In Line 2 and Line 1, the junction markers perfectly cosegregated with kanamycin resistance and were found in 75% of F2 sibs, confirming the linkage of the markers at a single locus. Segregation analysis in Line 9 suggested two independent transgenic loci, only one of which co-segregated with the junction marker.

As shown in Supplemental Figure 1 below, the transgene cluster in Line 1 integrated in a gene-rich region of chromosome 1 within the interval represented by BAC clone T30F21. In Line 2, the transgene cluster was located on chromosome 5 in a transposon-rich region within BAC clone F14I23 (Figure S1B). Line 9 contains two unlinked transgene loci, one of which co-segregated with the junction clone obtained by inverse PCR. The latter insertion mapped to the bottom of chromosome 4, within BAC clone T18B16, in a gene-rich region near the agamous (AG) gene (not shown).
